# Simultaneously integrated boost (SIB) spares OAR and reduces treatment time in locally advanced cervical cancer

**DOI:** 10.1120/jacmp.v17i5.6123

**Published:** 2016-09-08

**Authors:** Christine H. Feng, Yasmin Hasan, Malgorzata Kopec, Hania A. Al‐Hallaq

**Affiliations:** ^1^ Pritzker School of Medicine, The University of Chicago Chicago; ^2^ Department of Radiation and Cellular Oncology The University of Chicago Chicago IL USA

**Keywords:** SIB, cervical cancer, IMRT, PET‐avid lymph nodes

## Abstract

We performed a dosimetric comparison of sequential IMRT (sIMRT) and simultaneously integrated boost (SIB) IMRT to boost PET‐avid lymph nodes while concurrently treating pelvic targets to determine the potential of SIB IMRT to reduce overall treatment duration in locally advanced cervical cancer. Ten patients receiving definitive radiation therapy were identified retrospectively. RTOG consensus guidelines were followed to delineate the clinical target volume and organs at risk (OAR), which were then expanded per IMRT consortium guidelines to yield the planning target volume (PTV). Dosimetric parameters for PTVs and OAR including conformity (CI95%) were collected and compared using Wilcoxon signed‐rank tests with Bonferroni correction. The median PTV volume was 1843 cc (1088–2225 cc) and the median boost volume was 43 cc (15–129 cc). Comparable target volume coverage was achieved with sIMRT and SIB plans, while hot spots were significantly reduced using SIB. SIB plans improved sparing for all OAR, though only rectum and small bowel doses were statistically significant. Comparing sIMRT and SIB plans averaged over all patients, rectal doses were V45: 70.8% vs. 64.5%(p=0.002) and 0.1 cc: 50.7 Gy vs. 48.7 Gy (p=0.006). For small bowel, sIMRT and SIB IMRT plans yielded V45: 13.4% vs. 11.4%(p=0.006) and 1 cc: 54.4 Gy vs. 52.6 Gy (p=0.006), respectively. Doses to femoral heads and bladder trended towards significance in favor of SIB plans. The mean treatment time was 25 versus 29 days for SIB and sIMRT plans, respectively. When compared to sIMRT, SIB for treatment of nodal targets provides a significant, but small, dose reduction (3.8%–4.4%) to OAR, which leads to comparable biological dose despite higher fractional doses. Furthermore, SIB IMRT reduces overall treatment time and simplifies the planning process, and should be considered for targeting PET‐positive nodal disease in patients with locally advanced cervical cancer.

PACS number(s): 87.19.xj cancer

## I. INTRODUCTION

Cervical cancer is the third most common gynecologic cancer, with over 11,000 new diagnoses leading to over 3,900 deaths annually in the United States.^(1)^ For locally advanced disease, concomitant cisplatin‐based chemoradiation is considered the standard of care for definitive treatment of intact cervical cancer. However, severe acute and late gastrointestinal and genitourinary toxicities are a significant concern.^2^, ^3^ Intensity‐modulated radiation therapy (IMRT) for locally advanced cervical cancer has emerged as an advantageous technique for external beam radiation therapy. IMRT delivers radiation while minimizing doses to nearby organs at risk (OAR), such as the small bowel, bladder, rectum, and bone marrow.^4^, ^5^, ^6^, ^7^

NCCN guidelines recommend treatment of intact cervical cancer with external beam radiotherapy (EBRT) to 45 Gy in conventional fractionation (1.8–2 Gy), with a brachytherapy boost to the cervix and uterus prescribed such that accumulated dose from EBRT and brachytherapy to point A reaches ≤ 80 Gy.^(8)^ According to NCCN, EBRT doses of 10–15 Gy may be considered for adenopathy. Thus, patients with lymph node involvement often receive additional external beam boost doses to the nodal volumes.^(9)^ This sequential external beam boost^10^, ^11^ would extend the total treatment time, particularly if delivered prior to brachytherapy, and could also have potential dosimetric disadvantages with respect to dose to surrounding normal structures due to the additional boost dose. In contrast, simultaneously integrated boost (SIB) IMRT schemes deliver the initial and boost doses together in a smaller number of fractions, resulting in a shorter overall treatment time. Many retrospective studies, including one from our own institution,^(12)^ have shown that extended treatment time decreases pelvic disease control in this patient population.^13^, ^14^, ^15^

A study comparing sequential IMRT (sIMRT) and SIB IMRT treatment plans in various cancers revealed a marked increase in sparing of critical structures and increased conformity to target volumes.^(16)^ This suggests that SIB IMRT may deliver smaller doses to nontarget tissues, thus reducing toxicity to organs at risk during pelvic irradiation. A previous dosimetric comparison of sequential versus SIB IMRT plans in locally advanced vulvar carcinoma patients indicate that higher doses may be delivered to target volumes using SIB IMRT plans with no significant increase in radiation to surrounding organs.^(17)^ Thus, we performed a paired dosimetric comparison of sIMRT and SIB IMRT to treat the pelvic targets while either sequentially or simultaneously boosting PET avid lymph nodes in locally advanced cervical cancer.^11^, ^18^ Our goal was to assess dose to target volumes and organs at risk to determine the potential of SIB IMRT to reduce overall treatment duration.

## II. MATERIALS AND METHODS

### A. Patient identification

Data from all patients with intact cervical cancer and PET‐avid lymph nodes treated from 2009–2014 were retrospectively collected under institutional review board approval. All eligible 10 patients underwent CT simulation, which encompassed volumes from the sixth thoracic vertebral body to 5 cm below the femoral heads using CT slices of 3 mm thickness. Patients were simulated using contrast, as shown in Table 1, and treated in the supine position with customized alpha cradles. Patients (n=4) treated more recently were simulated under empty and full bladder conditions to delineate a PTV that covered the uterus while accounting for organ motion. For patients (n=6) treated with a sequential boost, a second simulation was performed 7–10 days prior to boost irradiation.

The Radiation Therapy Oncology Group (RTOG) consensus guidelines were followed for delineation of the clinical target volume (CTV) and organs at risk. The OAR contoured included the rectum, small bowel, bladder, femoral heads, and bone marrow. The target volumes included the gross tumor, cervix, uterus, parametria, upper portion of the vagina, and regional lymph nodes. For this study, boost volumes consisting of the PET‐avid regional lymph nodes, were contoured on the original pretreatment CT scan for SIB plans. Expansion of the nodal CTV by a 7 mm margin and expansion of the cervical CTV by a 15 mm margin, per IMRT consortium guidelines, yielded the PTV.^19^, ^20^ All contours were approved by a gynecologic radiation oncologist.

**Table 1:** Patient and treatment characteristics.

*Characteristic*	*Patients* (N=10)
Median Age	49
Range	29–65
*Stage*	
IB	2
IIA	1
IIB	4
IIIB	3
*Nodal Boost Location*	
Pelvic only	7
Para‐aortic only	2
Pelvic & Para‐aortic	1
*Target Doses and Volumes*	
Pelvic Dose	45 Gy
Median Boost Dose	9.5 Gy
Range	5.4–10.8 Gy
Median PTV Volume	909 cc
Range	1088–2351 cc
Median Boost Volume	43 cc
Range	15–135 cc
*Concurrent Chemotherapy*	
Cisplatin	10
*Immobilization*	
Upper and lower alpha‐cradles	10
*Image Guidance*	
kV	10
CBCT	4
*Simulation Contrast*	
Intravenous (IV)	6
Oral	10
Rectal	10
Bladder	10

### B. Treatment planning

Pinnacle (Version 9.0‐9.2; Philips Medical System, Milpitas, CA) was used to generate all treatment plans. Inverse‐planned IMRT calculations used seven to nine static fields with photon beams of 6 MV. A heterogeneity correction was applied for dose calculations and contrast in bladder, rectum, and small bowel was overridden to a density of 1.0 for all patients. All patients received a dose of 45 Gy in 25 fractions of 1.8 Gy to the pelvic PTV. Boost doses were limited by doses to OAR and ranged from 5.4–10.8 Gy (median=9.5 Gy), but remained the same for both sIMRT and SIB plans generated for each patient to enable paired comparisons. For example, an sIMRT plan generated to treat the pelvic PTV to 45 Gy with a nodal boost to 10 Gy in a total of 30 fractions was compared to an SIB plan treating the targets to the same doses in 25 fractions (i.e., 1.8 Gy per fraction to the pelvic PTV and 2.2 Gy per fraction to the boost PTV). Plans were optimized to achieve at least 95% coverage of the PTV with 95% of the prescribed dose while minimizing the volume that received more than 110% of the prescribed dose and maximally sparing the OAR, including small bowel, bladder, rectum, and bone marrow.

Composite doses for sIMRT and SIB plans were consistently assessed on the initial planning CT scan despite the fact that patients treated with a sequential boost received a second simulation CT scan. To accomplish this, the boost PTV was delineated on the sequential CT scan and its treatment beams were rigidly registered (Pinnacle 9.0–9.2) to the initial CT scan to enable dose summation for sIMRT plans. SIB plans were developed using only the initial CT scans. For all other patients who had one CT simulation scan, sIMRT and SIB plans were developed and calculated on this initial CT scan. Following EBRT completion, patients received brachytherapy with either LDR to 30 Gy in a single insertion or using HDR in five insertions per ABS guidelines.^13^, ^21^

### C. Data analysis

Dosimetric parameters were collected for the PTVs and OAR for metrics defined in the INTERTECC protocol.^(22)^ The conformity ratio (CI95%), defined as the ratio of the 95% dose volume to the PTV volume, was also collected for the pelvic and boost PTVs. For each patient, matched pairs of treatment plans (i.e., sIMRT and SIB) were compared using a paired nonparametric test. Wilcoxon signed‐rank tests (JMP‐version 9; Cary, NC) at the 5% significance level were used for all statistical comparisons. Bonferroni correction was used to account for multiple comparisons; the p‐value of 0.05 was divided by the number of comparisons made for each single organ, as calculated in Table 2.

The biological dose equivalent to 2 Gy fractions (EQD2) was also calculated for maximum dose points for OAR, using the equation:^(23)^

(1) $$EQD2=D\times \left[\frac{d+\alpha /\beta }{2+\alpha /\beta }\right]$$

where *D* is the total physical dose, *d* is the physical dose per fraction, and α/β=3 as recommended by the ABS.^(13)^ Wilcoxon signed‐rank tests at the 5% significant level were used to compare EQD2 among paired plans as described previously.

**Table 2:** Physical doses to target volumes and OAR for both sIMRT and SIB plans.

	*Sequential IMRT (mean)*	*SIB IMRT (mean)*	*p‐value*
*Pelvic PTV*			p<0.05/5=0.01
V90%	99.9%	99.9%	0.102
V95%	99.7%	99.4%	0.014
V110%	20.2%	7.5%	0.001^a^
V115%	9.3%	5.4%	0.002^a^
CI95%	0.996	0.988	0.115
*Boost PTV*			p<0.05/5=0.01
V90%	100%	100%	0.500
V95%	99.6%	100%	0.625
V110%	0.0%	0.0%	1.000
V115%	0.0%	0.0%	1.000
CI95%	0.996	1.000	0.264
*Rectum*			p<0.05/6=0.008
Mean	44.0 Gy	43.0 Gy	0.020
2 cc	49.7 Gy	47.3 Gy	0.006^a^
1 cc	50.0 Gy	47.6 Gy	0.006^a^
0.1 cc	50.7 Gy	48.7 Gy	0.002^a^
V30	94.5%	94.8%	0.770
V45	70.8%	64.5%	0.002^a^
*Bladder*			p<0.05/6=0.008
Mean	43.2 Gy	42.4 Gy	0.065
2 cc	49.2 Gy	48.0 Gy	0.020
1 cc	49.5 Gy	48.2 Gy	0.016
0.1 cc	50.1 Gy	48.6 Gy	0.014
V30	95.3%	95.2%	0.695
V45	58.4%	53.7%	0.027
*Small Bowel*			p<0.05/7=0.007
Mean	25.4 Gy	25.1 Gy	0.375
*250 cc*	37.1 Gy	36.3 Gy	0.193
2 cc	53.3 Gy	51.2 Gy	0.010
1 cc	53.7 Gy	51.6 Gy	0.006^a^
0.1 cc	54.4 Gy	52.6 Gy	0.010
V30	32.6%	31.4%	0.625
V45	13.4%	11.4%	0.006^a^
*Bone Marrow*			p<0.05/6=0.017
Mean	28.6 Gy	27.9 Gy	0.084
V10	91.6%	91.2%	0.981
V20	78.9%	78.0%	0.625
*Femoral Heads*			p<0.05/6=0.017
Mean	16.4 Gy	15.6 Gy	0.285
*0.1 cc*	44.6 Gy	46.1 Gy	0.006^a^
V30	11.6%	11.7%	1.000

^a^ Dosimetric parameters were significantly different between plans.

## III. RESULTS

**Figure 1:**
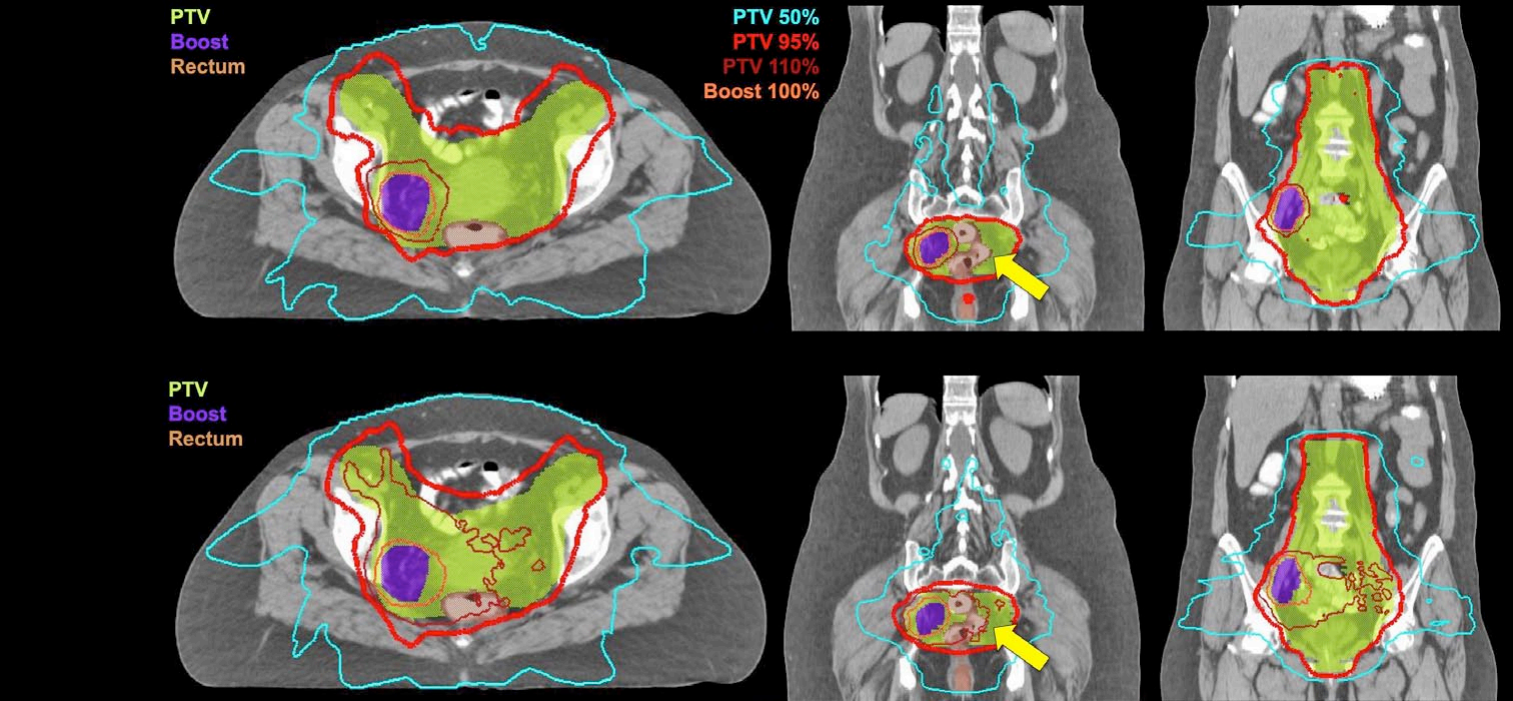
Dose distribution in axial and two coronal views obtained by sIMRT and SIB IMRT plans. SIB provides equally conformal (CI95%) and more homogenous doses (see V95%, V110%) to target volumes (colorwash) while sparing OAR (see yellow arrow).

Dosimetric parameters collected for each volume are listed in Table 2. There was no significant change in nodal boost volume between the initial and boost simulation scans (p=0.137). Comparable pelvic target volume coverage (V90% and V95%) was achieved with sequential boost and SIB plans, while hot spots (V110% and V115%) were significantly reduced using SIB. Coverage of boost target volumes was similar in both treatment groups, with little dose heterogeneity (Fig. 1).

**Figure 2:**
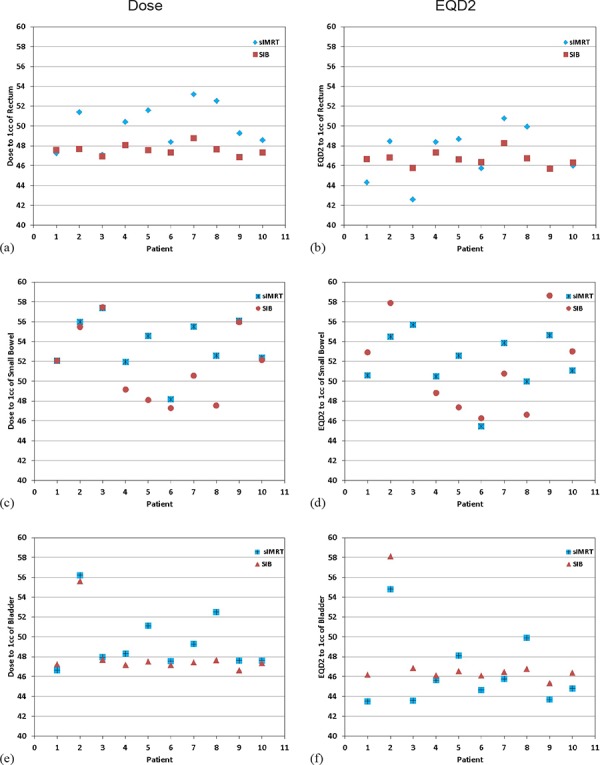
Scatter plots of physical dose and EQD2 to 1 cc of the rectum ((a), (b)), small bowel ((c), (d)), and bladder ((e), (f)) for each patient's sIMRT (blue markers) and SIB plan (red markers). For each patient, SIB significantly reduced 1 cc physical dose to the rectum and small bowel but not the bladder. For all OAR, EQD2 was comparable for both sIMRT and SIB plans despite the larger fractional dose delivered by SIB to nodal targets.

**Figure A1:**
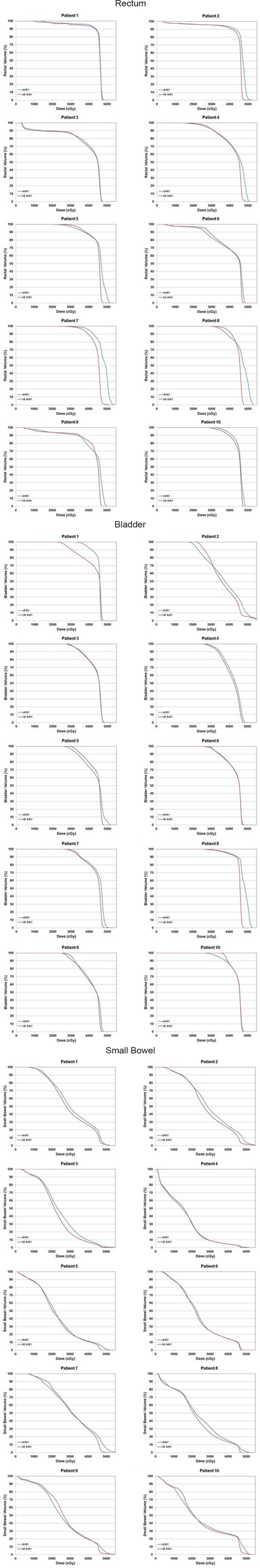
Individual dose‐volume histogram (DVH) plots of physical dose to rectum, small bowel, and bladder for each patient's sIMRT (blue lines) and SIB plan (red lines). SIB plans provide more OAR sparing over sIMRT at the highest doses.

Sparing of surrounding organs at risk was improved using SIB plans, though only differences in rectum and small bowel doses were statistically significant. The parameters measuring high doses were most reduced between sequential and SIB groups, especially V45, and maximum doses to 2 cc, 1 cc, and 0.1 cc (see dose‐volume histograms in Fig. A1 in Appendix A). Statistical improvements in physical dose indicated that SIB matched or surpassed the dose metric from the sIMRT plan for each of the 10 patients studied, as shown in the plots on the left‐hand side of Fig. 2. Significant reductions in physical doses to 2 cc, 1 cc, and 0.1 cc of the rectum and small bowel ranged between 3.9%–4.8% when averaged among all patients.

The doses to the bladder trended towards significance in favor of SIB plans, again illustrating the largest difference at the highest doses (Fig. 2). Dose to bone marrow was comparable between plans, with a slight improvement in sparing from the SIB plans. There was a statistically significant increase in the 0.1 cc dose to femoral heads in SIB versus sequential IMRT. However, mean doses and V30 parameters for femoral head volumes showed no difference between treatment schemes.

Maximum doses to small volumes of the OAR recalculated to equivalent 2 Gy fractions are listed in Table 3. Although physical doses to 2 cc, 1 cc, and 0.1 cc of certain OAR were significantly reduced by SIB (Table 2), there were no statistically significant differences in EQD2, with the exception of dose to 0.1 cc of the femoral heads (Table 3). Figure 2 depicts EQD2 (right‐hand side) for each of the 10 patients next to the physical dose plots (left‐hand side) for 1 cc to the rectum, small bowel, and bladder. These plots demonstrate that, even though physical doses were significantly reduced by SIB for the rectum and small bowel (first and second rows), EQD2 is comparable for both plan types. The median treatment time was 25 versus 30 days for SIB and sIMRT plans, respectively.

**Table 3:** Biologically equivalent doses in 2 Gy fractions (EQD2) for maximum point doses to OAR (α/β=3) for both sIMRT and SIB plans.

	*Sequential IMRT (mean)*	*SIB IMRT (mean)*	*p‐value*
*Rectum*			
2 cc	46.7 Gy	46.2 Gy	0.51
1 cc	47.1 Gy	46.7 Gy	0.51
0.1 cc	48.0 Gy	48.1 Gy	0.58
*Bladder*			
2 cc	46.1 Gy	47.3 Gy	0.09
1 cc	46.5 Gy	47.5 Gy	0.11
0.1 cc	47.3 Gy	48.1 Gy	0.28
*Small Bowel*			
2 cc	51.4 Gy	51.7 Gy	0.80
1 cc	51.9 Gy	52.3 Gy	0.65
0.1 cc	52.9 Gy	53.8 Gy	0.39
*Femoral Heads*			
0.1 cc	40.5 Gy	44.8 Gy	0.005^a^

^a^ Dosimetric parameters were significantly different between plans.

## IV. DISCUSSION

Previous studies have illustrated the improved conformity and homogeneity during delivery of radiation therapy using SIB IMRT over sequential IMRT in a variety of anatomic sites.^16^, ^17^ While some authors have investigated the use of SIB for replacing the brachytherapy boost of gross disease,^(23)^ there is no thorough dosimetric analysis of these parameters for boosting nodal targets in locally advanced cervical cancer. This study compares the treatment plans generated using sequential and SIB IMRT in patients with varying nodal boost locations. We found that SIB IMRT, when compared to sequential IMRT, provides comparable target coverage while reducing the volumes of doses higher than the prescription to the PTV. Our study also demonstrated improved OAR sparing, particularly for high doses to small volumes of the rectum and small bowel.

A similar comparison of these treatment schemes in vulvar carcinoma patients yielded comparable sparing of nearby structures, with differences in favor of SIB IMRT for the small bowel.^(17)^ Our results are consistent with these previous findings, but show greater sparing of organs‐at‐risk, including those partially embedded within the target volumes. A recent study from Bern Hospital showed SIB to PET‐avid lymph nodes resulted in comparable two‐year, disease‐free survival and acute toxicities to previously published studies involving sequential IMRT for cervical cancer patients.^(24)^ However, the authors note a concern with risk of acute bowel injury and lack of supporting data for doses exceeding 50 Gy, particularly with SIB to para‐aortic regions. In contrast, Vargo et al.^(11)^ reported on a retrospective series of patients treated with extended field IMRT with concomitant nodal boost to a median dose of 55 Gy delivered in 25 fractions, and found the regimen was well tolerated and provided adequate nodal control. Verma et al.^(25)^ focused on duodenal data from patients treated with extended field SIB IMRT, which demonstrated that the rate of duodenal injury is associated with V55 and significantly increases as V55 exceeds 15 cm^3^. Alternatively, Poorvu et al.^(10)^ did not find any correlation between duodenal or other gastrointestinal toxicities and dose when nodal boosts of up to 65 Gy were delivered. We found the small bowel doses to 2 cc and 0.1 cc were actually lower in the SIB plans for almost all patients, including those with para‐aortic boost volumes. However, we acknowledge that proximity or overlap of the boost volume to surrounding normal tissue is an important factor to consider when choosing a treatment plan, as SIB IMRT would subject a larger volume of the organ at risk to higher doses of radiation. Additional clinical experience is needed to prospectively assess the translational value of this data and potential risk of bowel injury with a higher radiobiological dose delivered with SIB.

Organ motion and CTV regression during treatment are also important factors to consider when prescribing higher doses to a boost volume. Herrera et al.^(26)^ used weekly on‐board megavoltage CT to track target volume motion and regression during six weeks of treatment. The pelvic CTV volume decreased over six weeks for all patients, but did not result in underdosing except in the two patients whose uteri became retroverted during the course of treatment. Bladder and rectum volumetric change and movement between fractions were also shown to significantly contribute to CTV underdosage. Planned and delivered doses to OAR at the end of treatment were not statistically different when considering the entire cohort. These findings are particularly important for patients in which the boost PTV volume is adjacent to or involves an OAR. The investigators of this study did not study motion of the nodal CTV, however, because they acknowledged that little motion is expected for nodal volumes that track bony anatomy.

SIB delivers a lower dose to surrounding OAR, especially rectum and small bowel, despite boosting a similar nodal volume. We found that dose differences between sequential and SIB IMRT schemes occurred consistently at the highest dose values. This implies that SIB allows for improved control of hot spots and redistribution of higher doses away from organs at risk. It is difficult to control the overlap of hot spots when creating a sequential boost treatment plan after the initial treatment.^(16)^ Thus, the improved benefit provided by SIB to OAR likely lies in the nature of its prospective and inclusive planning.

Wilcoxon signed‐rank tests were used to provide a nonparametric comparison of the data pairs, which are not assumed to be normally distributed. We used a Bonferroni correction to a significance level of p<0.01 to provide the most conservative approach, given the small sample size. However, this reduces the power of our analysis to detect true differences between SIB and sequential IMRT plans, in particular with regard to the bladder DVHs. Using a less conservative level of significance of p<0.05, the differences in doses to various bladder parameters are also statistically significant.

There are some limitations to our findings. The small sample size and treatment characteristic variations among patients may affect generalizability. All patients with locally advanced cervical cancer and PET‐avid lymph involvement treated at our institution from 2009–2014, for a total of 10, were included in this study. Although there was considerable variability in the nodal target location of the patients and in the treatment planning techniques (i.e., four patients were treated with SIB), we found that sample size did not greatly diminish the power of our study to detect significant differences between the two treatment modalities due to the power of the paired statistical test. In other words, each patient's sIMRT plan was compared to its corresponding SIB plan, thereby controlling for variations in nodal target characteristics. All SIB plans were developed on the initial CT scan to control for the patients treated with SIB. The inherent differences in dose distributions from treatment plans created by two different users may also affect results. We attempted to standardize this aspect with the usage of common OAR dose restrictions and thorough review by a single radiation oncologist to ensure clinically acceptable treatment plans. There were only two planners and 40% of the patients were treated with SIB, thus the proportion of sIMRT to SIB plans developed by each planner was comparable (6:4 vs. 4:6). With the retrospective design, the second planner consistently developed plans with *a priori* information and attempted to outperform the first planner regardless of whether planning sIMRT or SIB. Despite this effort, the dosimetric metrics for sIMRT plans were consistently inferior for each of the 10 patients, as shown in Fig. 2. In fact, for all the dosimetric parameters in Table 2 which were significantly improved by SIB planning, SIB outperformed sIMRT in 90%–100% of cases that were distributed among patients.

Delivering dose to OAR at a higher fractional dose could potentially increase toxicity. Thus, we calculated biological doses equivalent to 2 Gy fractions for doses to small volumes

(i.e., 2 cc, 1 cc, 0.1 cc) of OAR for each sIMRT and SIB plan for all patients (Table 3). Our results demonstrate that the small dosimetric advantage afforded by SIB leads to equivalent EQD2 doses for rectum, small bowel, and bladder. This implies that toxicities to these OAR are expected to be comparable. In fact, this has already been verified in a clinical study by Vargo et al.^(11)^ which also demonstrated adequate control of nodal disease and comparable toxicities to OAR when treated to 55 Gy in 25 fractions. In the case of the femoral heads, EQD2 to 0.1 cc was significantly worse in our SIB plans. This may be due to the fact that the femoral heads are located out of the primary radiation field and it is difficult to control out‐of‐field point doses. Furthermore, this parameter was not explicitly included in the planning constraints.

SIB affords several workflow and clinical advantages. Our study demonstrates that doses of up to 10 Gy to nodal targets can be safely incorporated into IMRT plans treating pelvic PTVs to 45 Gy without increasing the biological dose to OAR. Not only could this streamline the planning process, allowing hot spots to be reduced as shown in our study, but this could reduce the overall treatment duration by 5 fractions. The reduction in treatment time is of particular clinical significance as previous work from our institution^(12)^ and others^13^, ^14^, ^15^ has demonstrated that protracted treatment duration compromises local control in this patient population. Because nodal targets are not expected to be greatly affected by organ motion^(26)^ and because modest doses are indicated for nodal targets by NCCN guidelines, this is an ideal scenario in which to harness the power of SIB planning. To our knowledge, this is the first study to demonstrate the advantages of SIB over sIMRT for boosting PET‐avid nodal targets in locally advanced cervical cancer in a systematic manner. In fact, our results which demonstrate that SIB is expected to produce comparable toxicity and adequate nodal control, have been verified in a single‐institutional clinical trial using SIB to boost nodal targets to 55 Gy while delivering 45 Gy to the pelvic PTV in 25 fractions.^(11)^ When coupled with the clinical data that SIB treatment of nodal targets is well tolerated, our study indicates that SIB planning warrants clinical adoption for the treatment of nodal targets in cervical cancer.

## V. CONCLUSIONS

Our study demonstrated a significant dosimetric advantage of SIB IMRT when compared to sIMRT for the external beam irradiation of cervical cancer targets, which reduced high doses to both OAR and PTVs. Although the magnitude of dose reductions to small volumes of the rectum and small bowel were small (3.8%–4.3%), this resulted in comparable biological doses despite the higher fractional dose to nodal targets. Given the dosimetric parity to surrounding organs at risk and decreased overall treatment time by 5 fractions, in addition to the decreased time needed for calculation and implementation of multiple treatment plans, SIB IMRT merits clinical adoption for treatment of locally advanced cervical cancer.

## ACKNOWLEDGMENTS

Preliminary versions of this study were presented at the 56th Annual Meeting of the American Society of Radiation Oncology in San Francisco, CA. Support for institutional review board approval was provided by The University of Chicago Comprehensive Cancer Center support grant P30CA014599.

## COPYRIGHT

This work is licensed under a Creative Commons Attribution 3.0 Unported License.

## Supporting information

Supplementary Material

## References

[1] Centers for Disease Control and Prevention . Cervical Cancer Statistics [Internet]. Atlanta, GA: CDC; 2012.

[2] Morris M , Eifel PJ , Lu J , et al. Pelvic radiation with concurrent chemotherapy compared with pelvic and paraaortic radiation for high‐risk cervical cancer. N Eng J Med. 1999;340(15):1137–43. 10.1056/NEJM199904153401501 10202164

[3] Chemoradiotherapy for Cervical Cancer Meta‐Analysis Collaboration . Reducing uncertainties about the effects of chemoradiotherapy for cervical cancer: a systematic review and meta‐analysis of individual patient data from 18 randomized trials. J Clin Oncol. 2008;26(35):5802–12. 19001332 10.1200/JCO.2008.16.4368 PMC2645100

[4] Mundt AJ , Mell LK , Roeske JC . Preliminary analysis of chronic gastrointestinal toxicity in gynecology patients treated with intensity‐modulated whole pelvic radiation therapy. Int J Radiat Oncol Biol Phys. 2003;56(5):1354–60. 12873680 10.1016/s0360-3016(03)00325-0

[5] Mell LK , Tiryaki H , Ahn K‐H , Mundt AJ , Roeske JC , Aydogan B . Dosimetric comparison of bone marrow‐sparing intensity‐modulated radiotherapy versus conventional techniques for treatment of cervical cancer. Int J Radiat Oncol Biol Phys. 2008;71(5):1504–10. 18640499 10.1016/j.ijrobp.2008.04.046

[6] Portelance L , Chao KS , Grigsby PW , Bennet H , Low D . Intensity‐modulated radiation therapy (IMRT) reduces small bowel, rectum, and bladder doses in patients with cervical cancer receiving pelvic and para‐aortic irradiation. Int J Radiat Oncol Biol Phys. 2001;51(1):261–66. 11516876 10.1016/s0360-3016(01)01664-9

[7] Heron DE , Gerszten K , Selvaraj RN , et al. Conventional 3D conformal versus intensity‐modulated radiotherapy for the adjuvant treatment of gynecologic malignancies: a comparative dosimetric study of dose‐volume histograms. Gynecol Oncol. 2003;91(1):39–45. 14529660 10.1016/s0090-8258(03)00461-x

[8] Koh W‐J , Greer BE , Abu‐Rustum NR , et al. Cervical Cancer, Version 2.2015. J Nat Compr Cancer Netw. 2015;13(4):395–404; quiz 404. 10.6004/jnccn.2015.0055 25870376

[9] Gaffney D , Erickson B , Jhingran A , et al. ACR Appropriateness Criteria advanced cervical cancer. US Department of Health and Human Research, Agency for Healthcare Research & Quality. Rockville, MD: AHRQ; 2010.

[10] Poorvu PD , Sadow CA , Townamchai K , Damato AL , Viswanathan AN . Duodenal and other gastrointestinal toxicity in cervical and endometrial cancer treated with extended‐field intensity modulated radiation therapy to paraaortic lymph nodes. Int J Radiat Oncol Biol Phys. 2013;85(5):1262–68. 23182395 10.1016/j.ijrobp.2012.10.004

[11] Vargo JA , Kim H , Choi S , et al. Extended field intensity modulated radiation therapy with concomitant boost for lymph node‐positive cervical cancer: analysis of regional control and recurrence patterns in the positron emission tomography/computed tomography era. Int J Radiat Oncol Biol Phys. 2014;90(5):1091–98. 25303889 10.1016/j.ijrobp.2014.08.013

[12] Song S , Rudra S , Hasselle MD , et al. The effect of treatment time in locally advanced cervical cancer in the era of concurrent chemoradiotherapy. Cancer. 2013;119(2):325–31. 22806897 10.1002/cncr.27652

[13] Viswanathan AN , Thomadsen B , American Brachytherapy Society Cervical Cancer Recommendations Committee, American Brachytherapy Society. American Brachytherapy Society consensus guidelines for locally advanced carcinoma of the cervix. Part I: general principles. Brachytherapy. 2012;11(1):33–46. 22265436 10.1016/j.brachy.2011.07.003

[14] Shaverdian N , Gondi V , Sklenar KL , et al. Effects of treatment duration during concomitant chemoradiation therapy for cervical cancer. Int J Radiat Oncol Biol Phys. 2013;86(3):562–68. 23561652 10.1016/j.ijrobp.2013.01.037

[15] Lanciano RM , Pajak TF , Martz K , Hanks GE . The influence of treatment time on outcome for squamous cell cancer of the uterine cervix treated with radiation: a patterns‐of‐care study. Int J Radiat Oncol Biol Phys. 1993;25(3):391–97. 8436516 10.1016/0360-3016(93)90058-4

[16] Dogan N , King S , Emami B , et al. Assessment of different IMRT boost delivery methods on target coverage and normal‐tissue sparing. Int J Radiat Oncol Biol Phys. 2003;57(5):1480–91. 14630288 10.1016/s0360-3016(03)01569-4

[17] Bloemers MC , Portelance L , Ruo R , Parker W , Souhami L . A dosimetric evaluation of dose escalation for the radical treatment of locally advanced vulvar cancer by intensity‐modulated radiation therapy. Med Dosim. 2012;37(3):310–13. 22317848 10.1016/j.meddos.2011.11.005

[18] Esthappan J , Mutic S , Malyapa RS , et al. Treatment planning guidelines regarding the use of CT/PET‐guided IMRT for cervical carcinoma with positive paraaortic lymph nodes. Int J Radiat Oncol Biol Phys. 2004;58(4):1289–97. 15001274 10.1016/j.ijrobp.2003.09.074

[19] Taylor A , Rockall AG , Reznek RH , Powell ME . Mapping pelvic lymph nodes: guidelines for delineation in intensity‐modulated radiotherapy. Int J Radiat Oncol Biol Phys. 2005;63(5):1604–12. 16198509 10.1016/j.ijrobp.2005.05.062

[20] Lim K , Small W , Portelance L , et al. Consensus guidelines for delineation of clinical target volume for intensity‐modulated pelvic radiotherapy for the definitive treatment of cervix cancer. Int J Radiat Oncol Biol Phys. 2011;79(2):348–55. 20472347 10.1016/j.ijrobp.2009.10.075

[21] Nag S , Chao C , Erickson B , et al. The American Brachytherapy Society recommendations for low‐dose‐rate brachytherapy for carcinoma of the cervix. Int J Radiat Oncol Biol Phys. 2002;52(1):33–48. 11777620 10.1016/s0360-3016(01)01755-2

[22] IRTOC. International evaluation of radiotherapy technology effectiveness in cervical cancer (INTERTECC) . Phase II/III clinical trial of intensity modulated radiation therapy with concurrent cisplatin for stage I‐IVA cervical carcinoma [Electronic version]. La Jolla, CA: IRTOC; 2011.

[23] Guerrero M , Li XA , Ma L , Linder J , Deyoung C , Erickson B . Simultaneous integrated intensity‐modulated radiotherapy boost for locally advanced gynecological cancer: radiobiological and dosimetric considerations. Int J Radiat. Oncol Biol Phys. 2005;62(3):933–39. 15936580 10.1016/j.ijrobp.2004.11.040

[24] Cihoric N , Tapia C , Krüger K , Aebersold DM , Klaeser B , Lössl K . IMRT with ^18^FDG‐PET/CT based simultaneous integrated boost for treatment of nodal positive cervical cancer. Radiat Oncol. 2014;9:83. 24661323 10.1186/1748-717X-9-83 PMC4014138

[25] Verma J , Sulman EP , Jhingran A , et al. Dosimetric predictors of duodenal toxicity after intensity modulated radiation therapy for treatment of the para‐aortic nodes in gynecologic cancer. Int J Radiat Oncol Biol Phys. 2014;88(2):357–62. 24411609 10.1016/j.ijrobp.2013.09.053

[26] Herrera FG , Callaway S , Delikgoz‐Soykut E , et al. Retrospective feasibility study of simultaneous integrated boost in cervical cancer using Tomotherapy: the impact of organ motion and tumor regression. Radiat Oncol. 2013;8:5. 23286694 10.1186/1748-717X-8-5 PMC3551799

