# Supplementary figures and images for: Simultaneously integrated boost (SIB) spares OAR and reduces treatment time in locally advanced cervical cancer
Source: J Appl Clin Med Phys. 2016 Sep 8;17(5):76–89. doi: 10.1120/jacmp.v17i5.6123 (PMC5874085; doi:10.1120/jacmp.v17i5.6123)

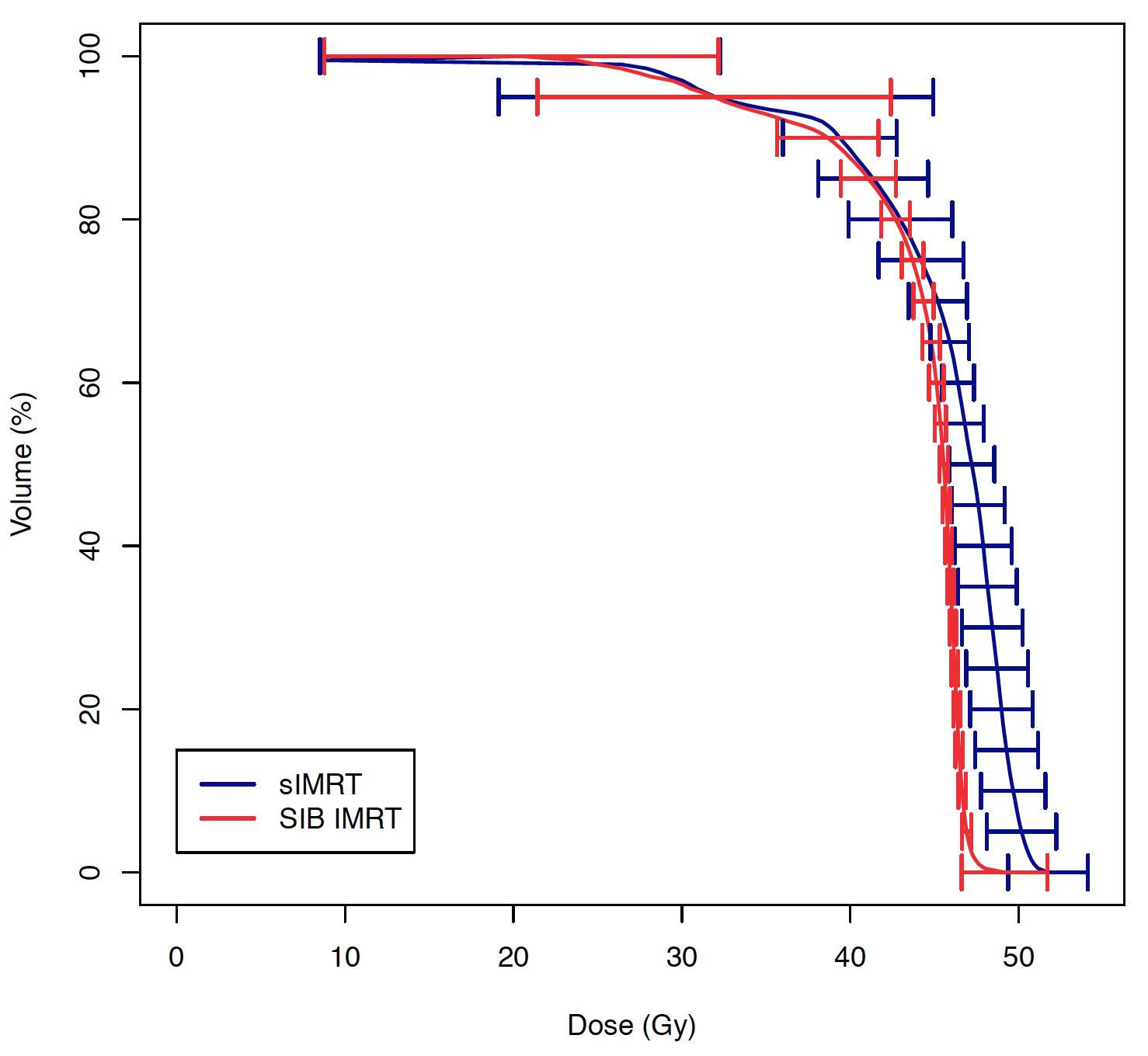

Supplement: Supplementary file 1 — Supplementary Material [file ACM2-17-076-s001.jpg]
